# New perspectives for natural antimicrobial peptides: application as antinflammatory drugs in a murine model

**DOI:** 10.1186/1471-2172-13-61

**Published:** 2012-11-17

**Authors:** Rosanna Capparelli, Francesco De Chiara, Nunzia Nocerino, Rosa Chiara Montella, Marco Iannaccone, Andrea Fulgione, Alessandra Romanelli, Concetta Avitabile, Giuseppe Blaiotta, Federico Capuano

**Affiliations:** 1 Faculty of Biotechnology, University of Naples “Federico II”, Naples, 80134, Italy; 2 Department of Biological Sciences, University of Naples “Federico II”, Naples, 80134, Italy; 3 Department of Food Science, University of Naples “Federico II”, Portici, 80055, Italy; 4 Department of Food Inspection IZS ME, via Salute 2, Portici, 80055, Italy

## Abstract

**Background:**

Antimicrobial peptides (AMPs) are an ancient group of defense molecules. AMPs are widely distributed in nature (being present in mammals, birds, amphibians, insects, plants, and microorganisms). They display bactericidal as well as immunomodulatory properties. The aim of this study was to investigate the antimicrobial and anti-inflammatory activities of a combination of two AMPs (temporin B and the royal jellein I) against *Staphylococcus epidermidis*.

**Results:**

The temporin B (TB-KK) and the royal jelleins I, II, III chemically modified at the C terminal (RJI-C, RJII-C, RJIII-C), were tested for their activity against 10 different *Staphylococcus epidermidis* strains, alone and in combination. Of the three royal jelleins, RJI-C showed the highest activity. Moreover, the combination of RJI-C and TB-KK (MIX) displayed synergistic activity. In vitro, the MIX displayed low hemolytic activity, no NO_2_^-^ production and the ability to curb the synthesis of the pro-inflammatory cytokines TNF-α and IFN-γ to the same extent as acetylsalicylic acid. In vivo, the MIX sterilized mice infected with *Staphylococcus epidermidis* in eleven days and inhibited the expression of genes encoding the prostaglandin-endoperoxide synthase 2 (COX-2) and CD64, two important parameters of inflammation.

**Conclusion:**

The study shows that the MIX – a combination of two naturally occurring peptides - displays both antimicrobial and anti-inflammatory activities.

## Background

Coagulase-negative staphylococci (CoNS) are highly abundant on the human skin, already a few hours after birth. The CoNS *Staphylococcus epidermidis* is an ubiquitous and permanent colonizer of human skin and the first cause of nosocomial infections [1]. Most infections with high morbidity and mortality are caused by methicillin-resistant strains of *Staphylococcus epidermidis* (MRSE) [2,3]. In addition, many MRSE strains form a capsule which favors biofilm development, where the pathogen can persist protected from antibiotics and invisible to the immune system [4,5].

New, unconventional antimicrobials are therefore urgently needed [6,7]. In this context, antimicrobial peptides (AMPs), in their natural form or after chemical modification, display interesting features as candidates to become new antimicrobials. They have a broad spectrum of activity against Gram-positive and Gram-negative bacteria, can be easily synthesized in laboratory and have limited toxicity for eukaryotic cells [8,9]. As innate immune components, AMPs lack specificity and immune memory, with the consequence that the pathogens rarely develop resistance to them [10]. Importantly, AMPs rapidly intercept and kill pathogens [11]. AMPs differ each other by size, sequence and secondary structure (α-helix or β-sheet) [12]. Most of them are hydrophobic and amphipathic [13]. AMPs can exert their activity by disrupting the membrane [14] or passing through the bacterial membrane [15]. Molecules belonging to the former class of AMPs permeabilize the membrane phospholipids bi-layer and kill the bacterial cell; those belonging to the latter class pass through the bacterial membrane and interacts with variable intracellular components, much as traditional antibiotics. AMPs, in addition to the antimicrobial activity, display also immune-modulatory properties (such as chemiotaxis, which contributes to bacterial elimination) and interact with natural and adaptive immunity [16,17]. Thus, in view of the above properties, AMPs represent one of the most promising future strategies for combating infections and microbial drug resistance. The present study describes two chemically modified AMPs - an analogue of the temporin B (TB-KK) secreted by the granular glands of the European red frog (*Rana temporaria*) [18] and an analogue of the royal jellein I (RJI-C) secreted by the mandible and hypopharyngeal glands of honeybees (*Apis mellifera*) [9,19]. These two peptides behave differently towards the bacterial membrane. RJI-C folds into beta sheets and aggregates onto the membrane; TB-KK folds into an alpha helix and does not aggregate onto the membrane [8,9].

Recent data demonstrate that hydrophobic peptides, when mixed with peptides possessing a net positive charge, give origin to a mixture with potential antibacterial activity [20,21]; second, that the combination of antimicrobial peptides derived from different organisms are highly active against Gram positive bacteria [9]. In agreement with these results, here we show that a mixture of TB-KK and RJI-C – two AMPs derived from different sources - displays strong antimicrobial activity against Gram-positive bacteria - modulates pro-inflammatory cytokines and nitric oxide production, in vitro and in vivo. The two peptides, following chemical modification, potentially can be made available in large quantities and in a homogeneous and highly pure form.

## Results

### Characterization of *Staphylococcus epidermidis* strains

**Figure 1:**
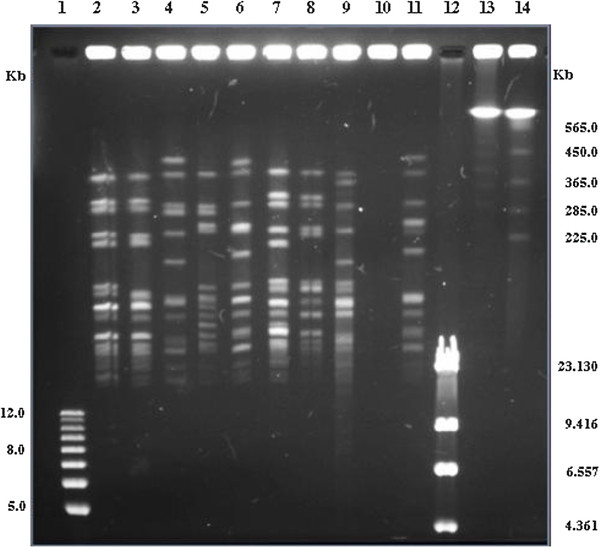
Sma I REA (Restriction Endonucleases Analysis)-PFGE patterns of Staphylococcus epidermidis strains: 1) 1Kb plus DNA Ladder (Invitrogen); 2) strain 5/25; 3) strain9/1; 4) strain 2/2; 5) strain 10/28; 6) strain 12/14; 7) strain 5/8; 8) strain 12/26; 9) strain 5/6; 10) strain SE (untypable); 11) strain 3/28; 12) Lambda DNA - Hind III Digested (Invitrogen); 13) DNA Size Standards - Lambda Ladder (Bio-Rad); 14) PFGE marker, 0.225–2.2 Mb S. cerevisiae chromosomal DNA (Bio-Rad).

To establish the clonal origin of the *Staphylococcus epidermidis* strains used in the study, the strains (10) were characterized phenotypically - with respect to their antibiotic resistance pattern and molecularly with respect to their Restriction Endonucleases Analysis (Pulse Field Gel Electrophoresis - REA-PFGE) pattern. All strains resulted resistant to aztreonam (30 μg; ATM30), bacitracin (10 μg; B2), cloxacillin (1 μg; CX1) and metronidazole (80 μg; M80) and sensitive to imipenem (10 μg; IPM10). The remaining 25 antibiotics displayed a strain specific pattern (Table 1). Also, with one exception (the strain SE), the strains displayed all different macro-restriction patterns, when analyzed by Sma I REA-PAGE (Figure 1). Thus, the strains used in this study belong to different clonal lineages.

**Table 1:** **Results of antibiotic susceptibility tests of ten S*taphylococcus epidermidis *strains**

**Antibiotics tested**																														
**Strain**	**FD10**	**P120**	**AMX25**	**AM10**	**ATM30**	**B2**	**CB100**	**CD30**	**FOX30**	**CAZ30**	**A30**	**CX1**	**K15**	**FF50**	**GM10**	**IPM10**	**MY2**	**M80**	**MZ75**	**NET30**	**FM300**	**NB30**	**T30**	**P10**	**PIP100**	**RF30**	**SP100**	**RL100**	**TE30**	**VA30**
SE	R	R	R	R	R	R	R	R	I	S	I	R	R	S	R	S	R	R	R	R	R	R	I	R	R	R	R	R	R	R
3/28	R	I	S	S	R	R	R	S	S	I	I	R	R	R	R	S	R	R	S	I	S	S	S	R	S	S	S	R	I	I
2/2	S	R	R	R	R	R	R	R	R	I	S	R	R	R	I	S	R	R	R	I	S	S	S	R	R	I	R	R	I	I
5/6	I	R	I	R	R	R	I	R	I	S	I	R	R	R	I	S	I	R	R	I	S	S	S	R	R	S	I	R	I	I
5/8	S	R	I	R	R	R	I	R	I	I	R	R	R	S	R	S	R	R	R	I	S	S	R	R	R	S	S	R	S	S
12/14	S	R	R	R	R	R	I	R	S	S	R	R	R	R	R	S	S	R	R	I	S	S	R	R	R	R	I	R	R	I
9/1	S	R	R	R	R	R	I	R	S	I	S	R	S	S	I	S	S	R	R	S	S	S	S	R	R	S	I	R	S	I
10/28	S	I	R	R	R	R	R	R	S	I	R	R	R	S	R	S	S	R	R	I	S	S	R	R	R	S	I	R	I	I
12/26	S	R	R	R	R	R	R	R	S	I	S	R	S	S	R	S	S	R	R	I	S	S	S	R	R	S	I	R	S	I
5/25	S	R	R	R	R	R	R	R	S	I	S	R	R	I	I	S	R	R	R	I	S	S	S	R	R	S	I	R	S	S

R= strain resistant to the antibiotic.

S= strain sensitive to the antibiotic.

I= intermediate strains sensitive to the antibiotic.

### In vitro antimicrobial activity of TB-KK and RJI-C

**Figure 2:**
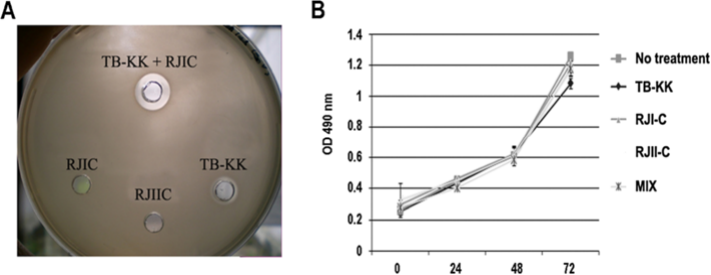
**(A) Antimicrobial activity of the single peptides (RJI-C 9 μg/ml; RJII-C 15 μg/ml; TB-KK 6 μg/ml) and of MIX (RJI-C at 9 μg/ml and TB-KK at 6 μg/ml) are shown as inhibition zone assay.** A larger zone of inhibition is evident around the MIX compared to the single components. (**B**) J774 cell line treated with the single peptides (RJI-C 9 μg/ml; RJII-C 15 μg/ml; TB-KK 6 μg/ml) or the MIX (RJI-C at 9 μg/ml and TB-KK at 6 μg/ml) maintain the same growth rate compare to the untreated control.

To evaluate the antimicrobial activity of RJI-C, RJII-C, RJIII-C and TB-KK (Table 2) these AMPs were tested in vitro [8,9], individually and in combination, against 10 *Staphylococcus epidermidis* strains. Among the three royal jelleins, RJI-C showed the highest activity (MIC: 30 μg/ml) (Table 3). Tested in various combination (RJI-C at 20 μg/ml and RJII-C at 5–20 μg/ml ; RJI-C at 20 μg/ml and RJIII-C at 5–20 μg/ml; RJII-C at 20 μg/ml and RJIII-C at 5–20 μg/ml), the royal jelleins did not display synergistic effects. Only RJI-C was thus tested for synergism with TB-KK. The combination of the two antimicrobials – RJI-C at 9 μg/ml and TB-KK at 6 μg/ml (MIX) – displayed a fractional inhibitory concentration index ≤ 0.5, which is evidence of synergism [20] (Table 3). The strains of *Staphylococcus epidermidis* were all sensitive to the MIX, but not its components (Table 4). This conclusion is supported by the larger inhibition ring of the MIX, compared to that of the individual components (Figure 2A).

**Table 2:** Peptide sequences and mass analysis of the royal jelleins (RJ) and temporin (TB) used in the study

**Peptide**	**Sequence**	**Calc. mass (DA)**	**Meas. mass (DA)**
RJI-C	PFKIDIHLGGY-NH_2_	1230.46	1231.02
RJII-C	TPFKISIHLGGY-NH_2_	1331.56	1331.90
RJIII-C	EPFKISIHLGGY-NH_2_	1359.57	1360.10
TB	YLLPIVGNLLKSLL-NH2	1391.80	1391.20
TB-KK.	KKYLLPI VGNLLKSLL-NH2	2295.40	2294.30

**Table 3:** The FIC index against Staphylococcus epidermidis strains: ≤ 0.5, synergy ; >0.5, no interaction

**Antimicrobial peptides**	**MIC**_**100**_	**Fic index**
RJI-C	30 μg/ml (24 μM)	
RJII-C	200 μg/ml (150 μM)	
RJIII-C	300 μg/ml (220 μM)	
TB-KK	7 μg/ml (3 μM)	
Gentamicin	5 μg/ml (10 μM)	
RJI-C + TB-KK	9 μg/ml + 6 μg/ml (7.3 μM + 2.6 μM)	0.5

**Table 4:** **Antimicrobial activity of the MIX and its components against different strains of *Staphylococcus epidermidis***

**Strains**	**% inhibition of bacterial growth RJI-C 9 μg/ml (7.3 μM)**	**% inhibition of bacterial growth TB-KK 6 μg/ml (2.6 μM)**	**% inhibition of bacterial growth RJI-C 9 μg/ml + TB-KK 6 μg/ml (RJI-C 7.3 μM + TB-KK 2.6 μM) (MIX)**
3/28	17 ± 2	19 ± 2	91 ± 1
2/2	18 ± 1	23 ± 0.5	96 ± 2
5/6	4 ± 3	10 ± 1	100 ± 0
5/8	12 ± 2	21 ± 2	95 ± 2
12/14	11 ± 0.5	20 ± 3	92 ± 1
9/1	18 ± 0	26 ± 2	96 ± 2
10/28	0	4 ± 1	100 ± 0
12/26	19 ± 2	14 ± 2	100 ± 0
5/25	15 ± 1	21 ± 1	90 ± 2

Interestingly, the antibacterial activity of the MIX against probiotics bacteria (*Lactobacillus plantarum*, *Lactobacillus Paracasei*, *Bifidobacterium animalis*) was five-fold lower than that of gentamicin (Table 5).

**Table 5:** Antimicrobial activity of the MIX or gentamicin on probiotic bacteria

**Strains**	**MIX RJI-C 9 μg/ml +TB-KK 6 μg/ml (RJI-C 7.3 μM +TB-KK 2.6 μM)**	**Gentamicin 5 μg/ml (10 μM)**
*Bifidobacterium animalis*	29% ± 3	96% ± 4
*Lactobacillum plantarum*	23% ± 2	97% ± 4
*Lactobacillum paracasei*	25% ± 2	96% ± 3

### In vitro hemolytic and cytotoxic activities of the MIX

To test the cytotoxic activity of the MIX, we used the hemolytic and the LC50 assays. The MIX lysed less than 12% of the murine erythrocytes (data not shown) and the LC50 value was 143,8 mg/ml versus 58.5 μg/ml of TB-KK and 64.6 μg/ml of RJI-C (Additional file 1: Table S1). The MIX was not toxic towards the macrophage J774 cells, which remained vital at 72 hours (Figure 2B).

### In vitro the MIX does not induce synthesis of NO^-^_2_

The MIX (RJI-C at 9 μg/ml and TB-KK at 6 μg/ml) did not induce NO_2_^-^ synthesis in J774 cells. Rather, when these cells were stimulated with LPS (10 μg/ml/well for 3 hours) and then treated with the RJ-IC, TB-KK and MIX reduced NO_2_^-^ synthesis (Table 6), one of the parameters to determine the cellular toxicity.

**Table 6:** **NO**_**2**_^**-**^**production of J774 cells: Mouse macrophages untreated, treated with RJI-C, TB-KK or the MIX, stimulated with LPS, stimulated with LPS and treated with RJI-C, TB-KK or the MIX**

**Treatment**	**Time of incubation (h)**		
	**24**	**48**	**72**
No treatment	0.25 ± 0.04	0.69 ± 0.02	0.92 ± 0.2
RJI-C (15 μg/ml) (12 μM)	0.42 ± 0.03	0.75 ± 0.01	1.02 ± 0.3
TB-KK (15 μg/ml) (6.5 μM)	0.82 ± 0.05	1.25 ± 0.2	1.34 ± 0.2
MIX (RJI-C 9 μg/ml + TB-KK 6 μg/ml) (RJI-C 7.3 μM + TB-KK 2.6 μM)	0.72 ± 0.3	0.85 ± 0.3	1.06 ± 0.2
LPS (10 μg/ml)	2.93 ± 0.2	10.96 ± 0.4	12.16 ± 0.5
LPS + RJI-C (15 μg/ml) (12 μM)	2.85 ± 0.3	8.42 ± 0.1	10.21 ± 0.2
LPS + TB-KK (15 μg/ml) (6.5 μM)	3.12 ± 0.6	9.75 ± 0.1	11.45 ± 0.2
LPS + MIX (RJI-C 9 μg/ml + TB-KK 6 μg/ml) (RJI-C 7.3 μM + TB-KK 2.6 μM)	2.63 ± 0.4	7.25 ± 0.3	8.26 ± 0.1

Data are expressed as micromoles of NO2- for 106 input cells, and are means ± standard deviation of three different experiments each performed in triplicate.

### In vitro anti-inflammatory activity of the MIX

**Figure 3:**
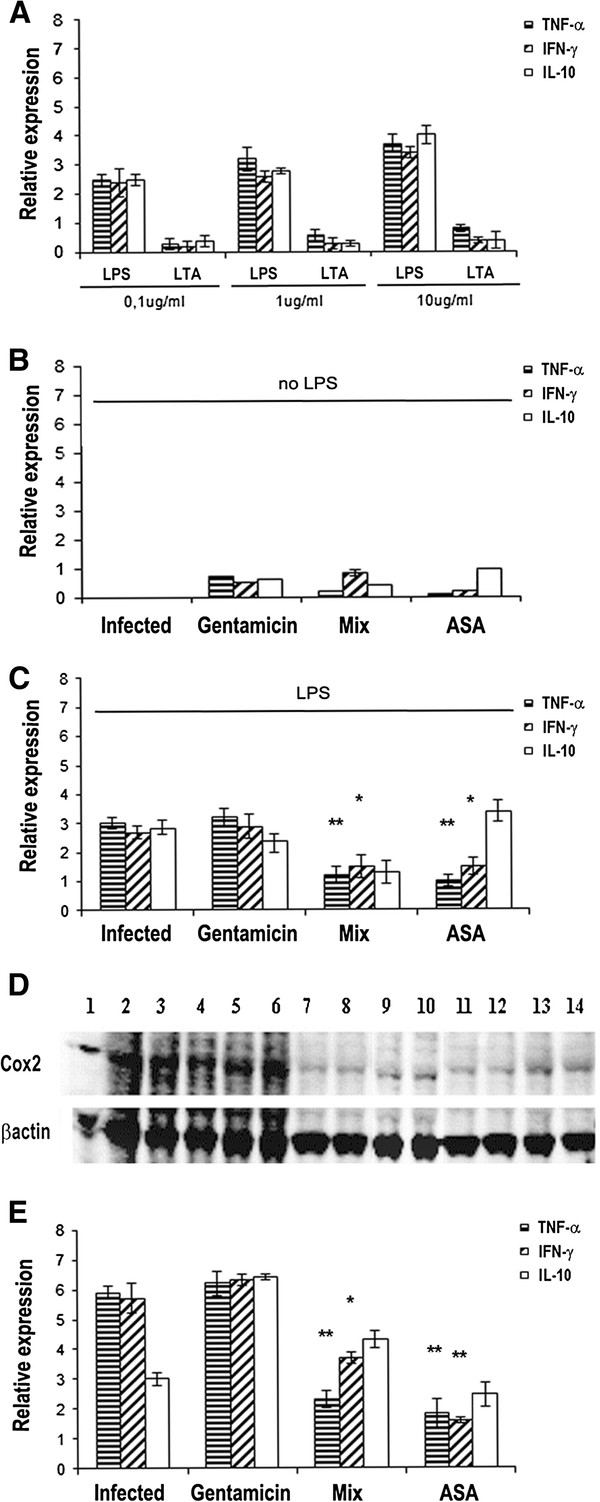
**(A) TNF-α, IFN-γ, IL-10 mRNA expression levels in J774 cells stimulated with LPS or LTA (0,1,1 or 10 μg/ml) for 3 hours.** (**B**) J774 cells treated with gentamicin (5 μg/ml) or MIX (RJI-C 9 μg/ml + TB-KK 6 μg/ml) or ASA (5 μg/ml) for 3 hours. (**C**) J774 cells stimulated with LPS (10 μg/ml) for 3 hours and treated with gentamicin (5 μg/ml) or MIX (RJI-C 9 μg/ml + TB-KK 6 μg/ml) or ASA (5 μg/ml) for further 3 hours. (**D**) Western blot analysis of COX-2 in J774 cell line. **Lane 1**–**3**: J774 cells + LPS(10 μg/ml); **Lane 4**–**6**: J774 cells + LPS (10 μg/ml) + inactive peptide (RJII-C 15 μg/ml); **Lane 7**–**9**: J774 cells + LPS (10 μg/ml) + ASA (5 μg/ml); **Lane 10**–**12**: J774 cells + LPS (10 μg/ml) + MIX (RJI-C 9 μg/ml + TB-KK 6 μg/ml; **Lane 13**–**14**: J774 cells + LPS (10 μg/ml) + gentamicin (5 μg/ml). (**E**) TNF-α, IFN-γ, IL-10 mRNA expression levels in kidney of mice (3mice/group) stimulated with LPS (250 μg, ~10 mg/Kg) for 3 hours; stimulated with LPS (250 μg, ~10 mg/Kg) for 3 hours and treated with gentamicin (5 μg/mouse) or MIX (RJI-C 9 μg/mouse + TB-KK 6 μg/mouse) or ASA (5 μg/mouse) for 3 hours. Values were normalized with GAPDH and compared to untreated control. *P <0.05, **p < 0.01; ***p < 0.001, Student’s *t* test gentamicin vs MIX and gentamicin vs ASA.

To investigate whether the MIX, in addition to the antimicrobial activity, also displays anti-inflammatory activity, J774 cells (10^6^ cells/well) were stimulated with either LPS or LTA (0.1, 1 or 10 μg/ml) for 3 hours. The results show that LPS stimulates inflammation in the J774 cells better than LTA (Figure 3A). Later, J774 cells were treated with gentamicin (5 μg/ml), acetylsalicylic acid (ASA, 5 μg /ml) or MIX (RJI-C 9 μg/ml + TB-KK 6 μg/ml) for 3 hours. In the absence of the agent causing inflammation (LPS), the MIX, gentamicin and ASA do not induce inflammation (Figure 3B). In J774 cells (10^6^ cells/well) stimulated with LPS for 3 hours, the MIX curbs the synthesis of the pro-inflammatory cytokines TNF-α and IFN-γ more efficiently than gentamicin and at the same extent of the ASA (Figure 3C).

These experiments demonstrate that the MIX exerts anti-inflammatory as well as antimicrobial activities, while the single components of the MIX have no anti-inflammatory activity (Additional file 2: Figure S1). Since COX-2 is a well-established parameter of inflammation [22] , the J774 cells were stimulated with LPS (10 μg/ml) and 1 hour later treated with the MIX, RJII-C (non-active peptide), acetylsalicylic acid (ASA), gentamicin or vehicle (PBS) for 3 hours. The level of the COX-2 protein was then detected by western blot. The MIX-treated cells, displayed a COX-2 protein level comparable to that of the cells treated with ASA or gentamicin, and much lower than that of the cells treated with RJII-C or the vehicle (Figure 3D). The above results demonstrate that the MIX curbs inflammation to the same extent as ASA [23].

### In vivo anti-inflammatory activity of the MIX in mice stimulated with LPS

To investigate further the property of the MIX to curb inflammation in vivo, LPS (250 μg, ~10 mg/Kg) was administrated to four groups of mice (3 mice/group). After 3 hours, the groups were treated respectively with the MIX (RJI-C 9 μg/mouse + TB-KK 6 μg/mouse), gentamicin (5 μg in 100 μl/mouse) or ASA (5 μg in 100 μl/mouse). The last group received 100 μl of saline buffer as control. After 3 hours, the mice that received the MIX showed a reduced level of both the pro-inflammatory cytokines TNF-α and IFN-γ, when compared to gentamicin-treated group, but an higher expression level of IFN- γ, when compared to the ASA group (Figure 3E). In conclusion, the MIX performs better than gentamicin, but worse than ASA.

### In vivo antimicrobial efficacy of the MIX given intravenously at 12 hours post infection

To evaluate the efficacy of the MIX to contrast microbial infection, four groups of mice (15 mice/group) were infected with lethal dose (10^8^ CFU/mouse) of *Staphylococcus epidermidis* (SE). This strain was chosen since it is resistant to the majority of the antibiotics tested (Table 1).

One group did not receive any treatment (control group); a second group received sterile PBS (100 μl/mouse) (placebo group – data not shown); the third group received the MIX (RJI-C 9 μg/mouse + TB-KK 6 μg/mouse); the fourth group received gentamicin (5 μg/mouse). PBS, MIX and gentamicin were administered intravenously at 3 hours post infection. In both, placebo and control groups, the bacterial load of kidneys and spleens increased progressively, while it decreased in the groups treated with gentamicin or the MIX (Additional file 3: Figure S2). Upon treatment of the mice with the MIX, the acute phase proteins, which represent important markers of inflammation [24], were evaluated (Additional file 4: Table S2). The SAA (Serum amyloid A), haptoglobin and fibrinogen were within normal ranges in the mice treated with the MIX or with gentamicin, while significantly high in the control mice (infected but not treated) (Additional file 4: Table S2).

### In vivo anti-inflammatory efficacy of the MIX given intravenously at 12 hours post infection

**Figure 4:**
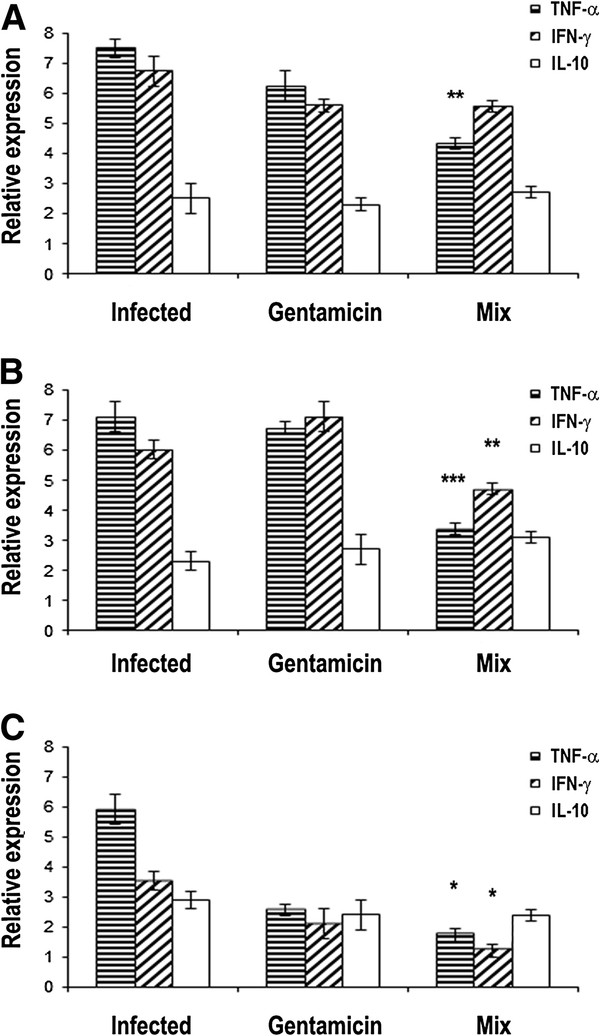
**(A-C) TNF-α, IFN-γ, IL-10 mRNA expression levels in infected mice with Staphylococcus epidermidis (108 CFU/mouse) or infected with Staphylococcus epidermidis (108 CFU/mouse) and treated with the MIX (RJI-C at 9 μg/mouse and TB-KK at 6 μg/mouse) or gentamicin (5 μg/mouse) at 3(A), 6 (B) and 9 (C) hours after treatment.** Values were normalized with GAPDH and compared to untreated control. *P <0.05, **p < 0.01; ***p < 0.001, Student’s *t* test gentamicin vs MIX.

The four groups of mice described before have been used also to evaluate the anti-inflammatory activity of the MIX. For this purpose, the expression levels of the TNF-α , IFN-γ, IL-10 cytokine genes were measured at 3, 6 and 9 hours after treatment in the kidney samples (Figure 4A-C, respectively). In the group treated with the MIX, the TNF-α and IFN-γ were under expressed (at 6, 9 hours from treatment), as compared to the group treated with gentamicin (Figure 4A-C). This result suggests that the MIX controls inflammation better than gentamicin.

**Figure 5:**
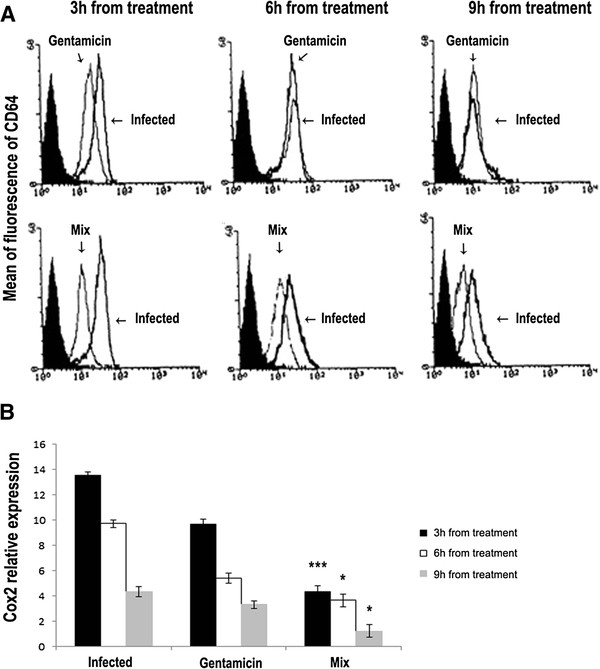
**(A) Using flow cytometry, CD64 levels were measured at 3, 6 and 9 hours after treatment in blood samples from mice infected with Staphylococcus epidermidis (108 CFU/mouse), from mice infected with Staphylococcus.epidermidis (108 CFU/mouse) and treated either with MIX (RJI-C at 9 μg/mouse and TB-KK at 6 μg/mouse) or with gentamicin (5 μg/mouse).** (**B**) mRNA expression level of COX-2, measured in kidneys of *Staphylococcus*.*epidermidis* (10^8^ CFU/mouse ) infected mice and in kidneys of *Staphylococcus epidermidis* (10^8^ CFU/mouse) infected mice and treated with MIX (RJI-C at 9 μg/mouse and TB-KK at 6 μg/mouse) or gentamicin (5 μg/mouse) . *p <0.05, **p < 0.01; ***p < 0.001, Student’s *t* test gentamicin vs MIX.

Also CD64 and COX-2 markers of inflammation in vivo were evaluated. Blood samples were collected 3, 6, or 9 hours after the treatments. CD64 was measured by flow cytometry (Figure 5A). Six and nine hours after the treatment with gentamicin or the MIX, the mice displayed a decreased expression of the CD64 marker (Figure 5A). The level of COX-2, was evaluated by RT-PCR on the mRNA extracted from kidney samples. In control mice displayed a significantly higher expression level of COX-2, compared to the mice treated with MIX or gentamicin. In the control mice COX-2 peaked 3 hours after the treatment. In the mice treated with gentamicin or the MIX, COX-2 expression level returned to the normal level nine hours after the treatment (Figure 5B).

**Figure 6:**
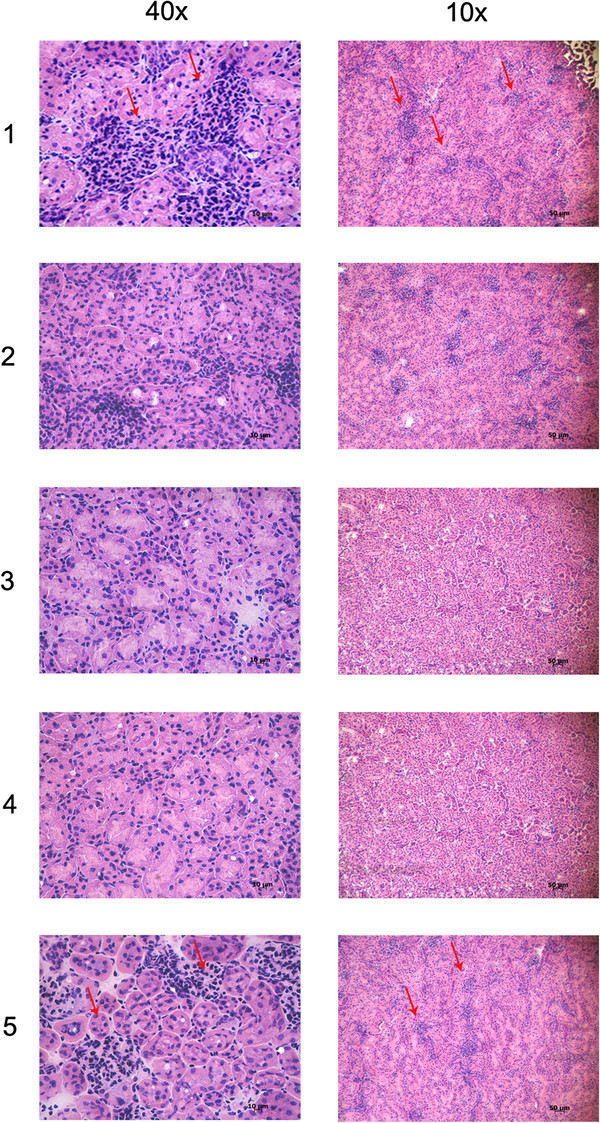
**Haematoxylin eosin staining.** Kidney sections from *Staphylococcus epidermidis* (10^8^ CFU/mouse) infected mice after 3 or 9 hours (panel 1and 5); kidney sections from *Staphylococcus epidermidis* (10^8^ CFU/mouse) infected mice after 3 hours and treated with MIX (RJI-C at 9 μg/mouse and TB-KK at 6 μg/mouse) for 3, 6 and 9 hours (panel 2–4).

To verify whether the MIX affected granulocytic infiltration in the kidneys of infected mice, hematoxylin-eosin staining was performed. As expected, kidneys of control mice displayed granulocytic infiltration within the lumen of the cortical convoluted tubules and hence lymphocytic infiltration, vessel activation and glomerular hyperplasia (Figure 6 panel 1, 5). Instead, kidneys of MIX-treated mice showed a dramatic reduction in the number of granulocytic cells localized in the cortical convoluted tubules, less glomerular hyperplasia, and no lymphocyte infiltration (Figure 6 panel 2–4).

### In vivo antimicrobial efficacy of the MIX for the period of 12 days

**Figure 7:**
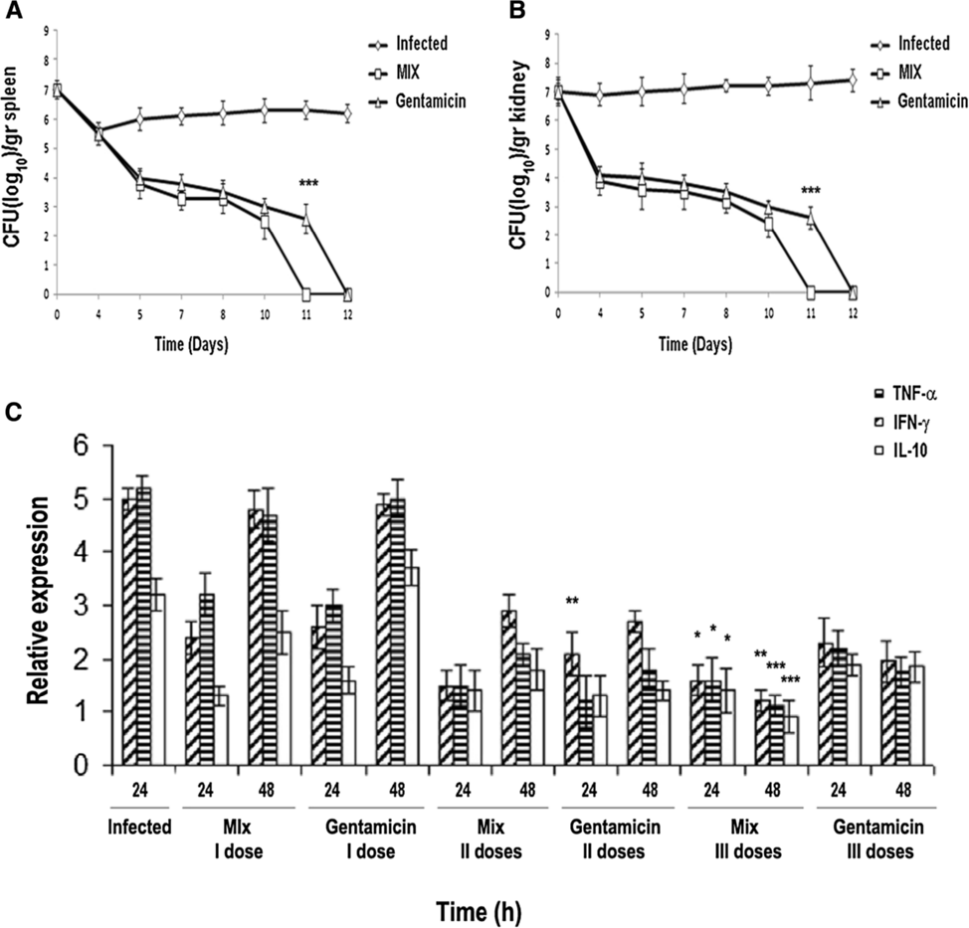
(A-B) Bacterial load in spleen and kidneys of animals (24/groups) infected with Staphylococcus epidermidis (107 CFU/mouse; rumble line); infected with Staphylococcus epidermidis(107 CFU/mouse) and treated with the MIX (RJI-C at 9 μg/mouse and TB-KK at 6 μg/mouse; square line) or gentamicin (5 μg/mouse; triangle line) *P <0.05, **p < 0.01; ***p < 0.001, Student’s t test gentamicin vs MIX. (**C**) TNF-α, IFN-γ, IL-10 mRNA expression levels were quantified, at the indicated time points, in mice infected with *Staphylococcus epidermidis* (10^7^ CFU/mouse) or infected with *Staphylococcus epidermidis* (10^7^ CFU/mouse) and treated with three different doses (I,II,III) of the MIX (RJI-C at 9 μg in 100 μl/mouse and TB-KK at 6 μg in 100 μl/mouse) or gentamicin (5 μg in 100 μl/mouse). Values were normalized with GAPDH and compared to untreated control. *P <0.05, **p < 0.01; ***p < 0.001, Student’s *t* test gentamicin vs MIX.

To test the antimicrobial activity of the MIX in vivo for a longer period, mice were infected with a sub-lethal dose (10^7^ CFU/mouse) of *Staphylococcus epidermidis* and then treated with the MIX. Four groups of mice (24 mice/group) were infected with the bacterial strain (SE). One group of mice did not receive any treatment (control group); a second group received sterile PBS (100 μl/mouse) (placebo group); the third group received the MIX (RJI-C: 9 μg/mouse + TB-KK: 6 μg/mouse); the fourth group received gentamicin (5 μg/mouse). PBS, MIX and gentamicin were administered intravenously in three boosts 3, 6 and 9 days post infection. In the placebo and the control groups, the bacterial load of kidneys and spleens (the target organs of the pathogen) increased progressively, while the load was significantly lower in the groups treated with gentamicin or the MIX. Eleven days after the infection, the mice treated with gentamicin were still infected, while those treated with the MIX were already sterile (Figure 7A-B).

Four days after the infection, in addition to spleen and kidneys (10^6^ CFU/gr and 10^7^ CFU/gr respectively), the bacterium was also detected (at a threshold level: 10^2^ CFU/g) in the liver (data not shown). Thus, the MIX is slightly more effective than gentamicin (Figure 7A-B). In all four groups, bacteria were no longer detected in the blood circulation within 2 h from infection (Additional file 5: Figure S3).

### In vivo anti-inflammatory efficacy of the MIX for the period of 12 days

To evaluate the anti-inflammatory activity of the MIX, the expression levels of the TNF-α, IFN-γ, IL-10 cytokines genes were measured in the kidneys. The experiment was carried out on the same four groups of mice described in the previous paragraph. For this purpose, the expression levels of the cytokines were measured 24 and 48 hours after each treatment with MIX (or 4, 5, 7, 8,10 and 11 days post infection). In the group treated with the MIX, compared to the group treated with gentamicin, the TNF-α and IFN-γ levels were under expressed (at 7 days) while the IL-10 levels were over expressed (at 10 days) (Figure 7C). This result suggests that the MIX controls inflammation better than gentamicin.

## Discussion

Recently we demonstrated that new antimicrobials are more effective than traditional antibiotics against *Staphylococcus epidermidis*[25,26]. The present study extends these results, providing evidence that the MIX – a mixture of a royal jellein modified at the C-terminal (RJI-C) and an analogue of temporin B (TB-KK) – is a valid alternative to the use of gentamicin against skin infections caused by *Staphylococcus epidermidis*.

In vivo, endogenous antimicrobial peptides (such as human defensins and cathelecidins) are known to be pleiotropic: they act as antimicrobials [27]; neutralize bacterial components (LTA and LPS), which otherwise would induce an excess of inflammation and tissue damage [28,29]; attract inflammatory cells to the wound site and promote wound healing.

The two exogenous components of the MIX also behave in a pleiotropic fashion: they control the bacterial load (Figure 7A-B and Additional file 3: Figure S2), inhibit the synthesis of pro-inflammatory cytokines (Figures 4 and 7C) and control the expression of COX-2 (Figures 3D and 5B), the acute phase proteins (Additional file 4: Table S2) and the expression of the CD64 receptor (Figure 5A). At the histological level, the MIX reduces kidney lymphocyte infiltration (Figure 6).

Mice infected with a sub-lethal dose of *Staphylococcus epidermidis* and three days later treated with the MIX (RJI-C: 9 μg/mouse + TB-KK: 6 μg/mouse), within 11 days from treatment, displayed sterile kidneys and spleen – the organs targeted by the bacterial strain used in this study (Figure 7A-B). Samples collected at 15 min intervals from infection showed that bacteria leave the blood circulation within 2 h (Additional file 5: Figure S3). These results are clinically relevant since they suggest that the MIX can potentially be used in humans, where infection is generally caused by a small initial inoculum and treatment is therefore initiated several days after infection (Figure 7A-B).

The MIX is not toxic for eukaryotic cells, in vitro and in vivo (Figure 2B); its components act synergistically (Figure 2A) and becomes moderately hemolytic (12%). In addition, the MIX reduces the synthesis of NO_2_^-^ in cells infected with *Staphylococcus epidermidis* (Table 6). These additional properties make the MIX a candidate for a new generation drug.

In vitro and in vivo experiments demonstrate that the MIX down regulates the level of the pro-inflammatory cytokines TNF−α and IFN-γ while enhancing the expression of the anti-inflammatory cytokine IL-10. This effect is comparable to that of gentamicin, a well-known antimicrobial drug. These results confirm that the MIX, in addition to an antibacterial activity, also exerts – in vivo and in vitro - an anti-inflammatory activity.

The intestinal flora represents a defense barrier against pathogens [30]. We therefore also investigated whether the MIX spared probiotic bacterial species in vitro. While gentamicin killed the totality of the probiotics tested (*Lactobacillus plantarum*, *Lactobacillus Paracasei*, *Bifidus animalis*), the MIX killed a minority of each bacterial species (29%-23%-25%, respectively) (Table 5).

The influence of the MIX on the major cell signaling pathways was also studied. CD64 and COX-2 warn about the cell exposure to inflammatory stimuli [31,32]. The MIX reduced the expression level of COX-2 (Figures 3D and 5B) and CD64 (Figure 5A), proofing that the MIX exerts also anti-inflammatory activity. The CD64 levels are high in the mice infected. In the mice infected and then treated with MIX at both 3, 6 and 9 hours from treatment, levels of CD64 are reduced (Figure 5A). This last result provides evidence that the MIX has effects on mechanisms of both innate and adaptive immunity.

## Conclusions

This study provided evidence which suggests an analogy between endogenous AMP and the MIX, consisting of exogenous and chemically modified AMPs. Both display a two-fold role, rapidly recognizing the presence of a pathogen and preventing an excess of inflammation.

## Methods

### Bacteria

List and origin of *Staphylococcus epidermidis* used in this study is reported in Additional file 6: Table S3. All strains were isolated from patients hospitalized at the Medical School of the University of Naples Federico II. All strains were molecular identified by means of *kat* A-RFLP analysis technique described by Blaiotta et al. [33].

The study does not investigate clinical aspects of the disease, nor it uses human specimen. The study therefore does not require the Ethic Committee approval.

### Antibiotic susceptibility of *Staphylococcus epidermidis* strains

The antibiotic-susceptibility profile of strains was tested using the disk diffusion method on Mueller-Hinton agar, according to the NCCLS guidelines (2002) [34]. The antibiotics used and their concentrations were as follows: amoxicillin (25 μg; AMX25), ampicillin (10 μg; AM10), aztreonam (30 μg; ATM30), bacitracin (10 μg; B2), carbenicillin (100 μg; CB100), ceftazidime (30 μg; CAZ30), cefoxidin (30 μg; FOX30), cephaloridine (30 μg; CD30), cloxacillin (1 μg; CX1), erythromycin (15 μg; E15), fosfomycin (50 μg; FF50), fusidic acid (10 μg; FD10), gentamicin (10 μg; GM10), imipenem (10 μg; IPM10), lincomycin (2 μg; MY2), metronidazole (80 μg; M80), mezlocillin (75 μg; MZ75), netilmycin (30 μg; NET30), nitrofurantoin (300 μg; FM300), novobiocin (30 μg, NB30), oxytetracycline (30 μg, T30), penicillin-G (10 μg; P10), piperacillin (100 μg, PIP100), rifampicin (30 μg; RF30), chlorotetracycline (30 μg; A30), spiramycin (100 μg; SP100), sulfamethoxazole (100 μg; SP100), tetracycline (30 μg; TE30), and vancomycin (30 μg; VA30). All antibiotics were provided by BioMérieux SA, (Marcy l’Etoile, France).

### Pulsed-field electrophoresis of *Staphylococcus epidermidis* strains

The procedure adopted was that described [35]. Briefly, inserts of intact DNA were digested in 200 μl of appropriate buffer supplemented with 40 U of *Sma* I (Promega, Milan). Pulsed field gel electrophoresis (PFGE) of the restriction digests was performed by using the CHEF system (Bio-Rad Laboratories, Hercules, CA, USA) with 1% (wt/vol) agarose gels and 0.5 x TBE as running buffer, at 10°C. Restriction fragments were resolved in a single run, at constant voltage of 6 V cm^2^ and an orientation angle of 120° between electric fields, by a single phase procedure for 24 h with a pulse ramping between 1 and 50s.

### Antibacterial activity of AMPs

Antibacterial activity of the peptides used in this work was evaluated as described previously [8]. A potential synergism (FIC) between TB-KK and RJI-C (MIX) was evaluated by adding combinations of two peptides in a serial two-fold dilutions (RJI-C 5–100 μg, 40 μl/well; TB-KK 5–100 μg, 40 μl/well;) to wells containing 10^5^ CFU/well in 60 μl [8]. The fractional inhibitory concentration (FIC) index for combinations of two peptides was calculated according to the equation: FIC index = FICA + FICB = A/MICA + B/MICB , where A and B are the MICs of drug A and drug B in the combination, MICA and MICB are the MICs of drug A and drug B alone, and FICA and FICB are the FICs of drug A and drug B. The FIC indices were interpreted as follows: ≤0.5, synergy; 0.51–4.0, no interaction; > 4.0, antagonism [23].

The growth inhibition percentages of *Staphylococcus epidermidis* and probiotic strains were assessed under the same conditions.

### Inhibition zone assay and test of the haemolytic activity of the antimicrobials

The MIX (RJI-C at 9 μg/ml and TB-KK at 6 μg/ml) was tested for its haemolytic activity using mouse red blood cells and for inhibition zone assay test [8]. The MIX was tested for its haemolytic activity using mouse red blood cells. The blood was collected from the tail of the animals and centrifuged (4x10^2^ g for 3 min). The erythrocytes were washed with saline, suspended at 3x10^6^ erythrocytes/ml, mixed with the peptide combination (RJI-C 9 μg and TB-KK 6 μg in 100 μl saline) and incubated for 1 h at 37°C. The haemolytic activity was measured according to the formula OD _peptide_ - OD _negative control_/OD _positive control_ - OD _negative control_ X 100 where the negative control (0% haemolysis) was represented by erythrocytes suspended in saline and the positive control (100% haemolysis) was represented by the erythrocytes lysed with 1% triton X100 [36].

The LC50 values relative to the two peptides and the MIX were calculated as described [37].

### Cell culture

J774 murine macrophages from the American Tissue Culture Collection (ATCC, Rockville, MD,USA) were cultured in Dulbecco's modified Eagle's medium (DMEM, Cambrex Bio Science, Verviers, Belgium). Culture media contained 10% fetal bovine serum (FBS, Sigma, Milan, Italy), 100 IU/ml penicillin, 100 μg/ml streptomycin (all from Gibco, Paisley, Scotland). Cells were seeded on 96-well plates (Falcon, Milan) for the MTT Assay, and on 24-well plates (Falcon, Milan) for NO_2_^−^ measurements, fluorescence microscopy analysis, and RT-PCR assays. Cell monolayers were grown to adherence before the experiments were started.

### Mice

Experiments were carried out on female BALB/c mice (aged 8 to 10 weeks) at the animal facility of the University of Naples. Bacteria (10^7^ or 10^8^ CFU/mouse) were inoculated by intravenous routes (i.v.). LPS (250 μg, ~10 mg/Kg) (Sigma-Aldrich Milan), or an equivalent volume of sterile 0,9% saline vehicle (250 μl) was administered intraperitoneally. Blood samples were drawn from the tail vein using 0.5 ml syringes. Spleen and kidney were collected at several time points (4,5,7,8,10, 11and 12 days) after the mice infection with a sub-lethal dose of *Staphylococcus epidermidis* (10^7^ CFU/mouse). However the same organs were also collected at 3, 6, 9 and 12 hours after infection with a lethal dose of *Staphylococcus epidermidis* (10^8^ CFU/mouse). Spleens and kidneys were dissected and weighed. One g of each sample was homogenized in 1 ml saline and serially diluted in saline.

Colony forming units (CFU) were evaluated by the plate count assay. Animal experiments were approved by the Animal Care Committee of the University of Naples.

### Measurement of cell viability

Analysis of cell viability was performed using the CellTiter 96® AQueous One Solution Cell Proliferation Assay system (MTS assay) (Promega, Madison,WI, USA). J774 cells were seeded at 2500 cells per well in a 96-well plate and incubated at 37°C, in a humidified atmosphere with 5% CO_2_. TB-KK 15 μg/ml, RJI-C 15 μg/ml, MIX (TB-KK 6 μg/ml + RJI-C 9 μg/ml) or RJII-C (Control 15 μg/ml) were added to the medium immediately after cell adhesion. At each time point 20 μl of CellTiter 96® AQueous One Solution reagent was added to each well, according to the manufacturer's instructions. Absorbance was recorded at 490 nm after 2 h using an EnVision 2102 multilabel reader (PerkinElmer, Waltham, USA).

### Nitrite formation in J774 cells stimulated with LPS and treated with RJI-C, TB-KK, and the MIX

Nitrite accumulation (NO_2_^−^, μmol/10^6^ cells) in the cell culture medium was determined by the Griess reaction [38].

### Western Blot Analysis COX-2

Cell lysates for Western blotting were prepared by washing cells twice with ice-cold phosphate-buffered saline followed by cell lysis in 500 μl of Fastprep lysis buffer (1X protease inhibitor cocktail tablet (Roche EDTA free) resuspended in 1X PBS) on ice and lysed 20s at 6.5 intensity, 2X intervalling with 5–10 minutes on ice. Cell lysates were centrifuged for 10 min at 7800 *g* at 4°C, and the supernatants were collected and stored at −80°C until analysis. Lysate protein concentrations were measured using the Bio-Rad protein assay method, as described in the manufacturer’s instructions. Cell lysate volumes corresponding to 20 μg of total protein were diluted 1:1 in Laemmli buffer (Bio-Rad) and boiled for 5 min prior to electrophoresis on a 10% acrylamide gel. The resolved proteins were electroblotted on PVDF membrane (Bio-Rad) by the Bio-Rad semidry transfer method, according to the manufacturer’s instructions. Membranes were stained with PonceauRed to verify uniform protein transfer, and then blocked with blocking buffer (1X TBS, 0.1% Tween-20, 5% w/v non-fat dry milk) for 1 h at RT. Blocked membranes were incubated overnight at 4°C with COX-2 mouse monoclonal antibody (diluted 1/2000), β-actin mouse monoclonal antibody (diluted 1/10,000). Blots were washed three times in TBS-Tween before incubation with the appropriate horseradish peroxidase-conjugated secondary antibody (sheep anti-mouse IgG diluted 1/5000) for 1 h at room temperature.

After three washes with TBS-Tween, the signal was developed using standard procedure. Gel image was acquired in Fujifilm LAS-3000 Chemiluminescence system (Fujifilm Life science).

### Real time PCR of pro-inflammatory

Total RNA was isolated from the tissue and the cell line after treatment by using Trizol reagent (Invitrogen, Milan, Italy). RNA was suspended in RNase-DNAse free distilled water, assessed for concentration (by measuring the absorbance at 260 nm) and purity (by ascertaining that the A260/A280 ratio was .1.9). RNA (1 μg) was then treated with 1U RNAse-free DNAse (Promega, Madison, WI). DNA contamination of RNA samples was excluded by PCR with primers specific for the gapdh gene. Reverse transcription was carried out with ImProm-II reverse transcriptase (Promega, Madison, WI) and oligo(dT). Real-time PCR was performed on 50 ng cDNA, using 1x master mix SYBRGreen (Applied Biosystem, Milan) in a StepOne Applied Biosystem instrument (Applied Biosystem, Milan). Reactions were performed in 20 μl in triplicate. The primer list is reported in Additional file 7: Table S4.

### ELISA test of pro-inflammatory cytokines

In addition, the ELISA test was used to measure the anti-inflammatory activity of the MIX and its components : RJI-C 9 μg/mL e TB-KK 6 μg/mL.

Briefly, J774 cells (10^6^ cells/well) were stimulated with LPS (10 μg/ml; 1 hour), treated with RJI-C 9 μg/ml or TB-KK 6 μg/ml or MIX (RJI-C 9 μg/ml + TB-KK 6 μg/ml) in presence or absence of LPS (10 μg/ml). The supernatants from these cells (100 μl/well) were transferred into the wells of a plate previously coated with mouse anti-human TNF-α (BD Pharmingen; 50 μl diluted 2 x 10-3/well) or mouse anti-human IFN-γ (Biosciences, 50 μl diluted 2 x 10-3/well) along with a second dose of anti IFN- γ or TNF- α, HRP-labelled rabbit anti mouse IgG diluted 10^-3^ (100 μl/well) and TMB peroxidase substrate (BIORAD; 100 μL/well), in the order. The optical density of each well was read at 405 nm using a microplate reader (Bio-Rad, Japan). Triplicate positive and negative controls were included in each plate [39].

### Cytofluorimetric analysis

CD64 expression in total White Blood Cells was analyzed using a Flow cytometry EPICS Elite (Beckman Coulter, Fullerton, CA). Daily instrument quality control including fluorescence standardization, linearity assessment, and spectral compensation were performed to ensure identical operation from day to day. At least 10.000 events for each sample was analyzed and the data were saved for later analysis on EXPO32 software (Beckman Coulter). Data analysis was performed by using electronic gating on the basis of FSC and SSC excluded cellular debris and nonviable cells. PE-coniugated anti-mouse CD64 expression was measured using a log_10_ scale. Briefly, 50 ul of whole blood was incubated for 10 minutes at room temperature with saturating amounts of phycoeritrine- conjugated anti-CD64 murine monoclonal antibody (Becton Dickinson) followed by red blood cell lysis with an ammonium chloride–based red cell lysis solution (Beckman Coulter, Fullerton, CA). Samples were then washed once and resuspended with phosphate-buffered saline at pH 7.4, to a volume of 1 mL.

### Other methods

The kidney was fixed in 10% buffered formalin, sectioned (10 μm) and stained with hematoxylin-eosin according to standard protocols. Bacterial counts and cytokine levels were analyzed using Student’s *t* test.

## Competing interests

The authors declare that they have no competing interests.

## Authors’ contributions

RC designed the study and wrote the paper. AR and CA designed and synthesized the peptides. GB carried out the antibiotic resistance test. NN, RCM and MI carried out cell culture and in vivo tests. FDC, AF and FC carried out biochemical, statistical and in vivo tests. All authors read and approved the final manuscript.

## Supplementary Material

Additional file 1 **Table S1.** Lethal concentration (LC_50_) of Temporin B –KK, Royal jelleins-IC, MIX through their hemolytic activity on mouse erythrocytes. Click here for file

Additional file 2 **Figure S1.** Anti-inflammatory activity. The levels of IFN- γ and TNF- α were determined by a sandwich ELISA test in J774 cells untreated; J774 cells infected with *S*. *epidermidis* for 1 h; J774 cells stimulated with RJI-C (9 μg/ml) for1 h; J774 cells stimulated with TB-KK. (6 μg/ml) for1 h; J774 cells stimulated with MIX (RJI-C 9 μg/ml + TB-KK 6 μg/ml) for1 h; J774 cells infected with *S*. *epidermidis* for 1 h and stimulated with MIX for 1 h. Results from two representative experiments are presented as mean value ± S.D. *P <0.05, **p < 0.01; ***p < 0.001, Student’s *t* test *S*. *epidermidis* vs *S*. *epidermidis* + MIX. Click here for file

Additional file 3 **Figure S2 (A-B).** Bacterial load in spleen and kidneys of *S.epidermidis* infected mice (rumble line) and subsequently treated with the MIX (square line) or gentamicin (triangle line). Data are representative of 15 animals/group. Student’s *t* test gentamicin vs MIX not significant. Click here for file

Additional file 4 **Table S2.** Acute phase proteins. Acute phase proteins from blood samples of mice infected with *Staphylococcus epidermidis* and treated with MIX or with Gentamicin. Click here for file

Additional file 5 **Figure S3.** Time course (30, 60, 90 and 120 minutes) of bacterial load in blood of *S*. *epidermidis* infected mice. Click here for file

Additional file 6 **Table S3:** Origin of S. epidermidis strains. Click here for file

Additional file 7 **Table S4:** Sequences of the primers. Click here for file
