# Supplementary material for: New perspectives for natural antimicrobial peptides: application as antinflammatory drugs in a murine model
Source: BMC Immunol. 2012 Nov 17;13:61. doi: 10.1186/1471-2172-13-61 (PMC3526545; doi:10.1186/1471-2172-13-61)
Supplement: Additional file 1 — Table S1. Lethal concentration (LC50) of Temporin B –KK, Royal jelleins-IC, MIX through their hemolytic activity on mouse erythrocytes. [file 1471-2172-13-61-S1.doc]

| **Peptides** | **Lethal concentration LC50** |
| --- | --- |
| Temporin B -KK | 58,52 µg/ml |
| Royal jelleins-IC | 64,75 µg/ml |
| MIX | 143,8 µg/ml |

**Table S1: Lethal concentration (LC_50_) of Temporin B –KK, Royal jelleins-IC, MIX** **through their hemolytic activity on mouse erythrocytes.**
