# Supplementary figures and images for: New perspectives for natural antimicrobial peptides: application as antinflammatory drugs in a murine model
Source: BMC Immunol. 2012 Nov 17;13:61. doi: 10.1186/1471-2172-13-61 (PMC3526545; doi:10.1186/1471-2172-13-61)

Figure S1

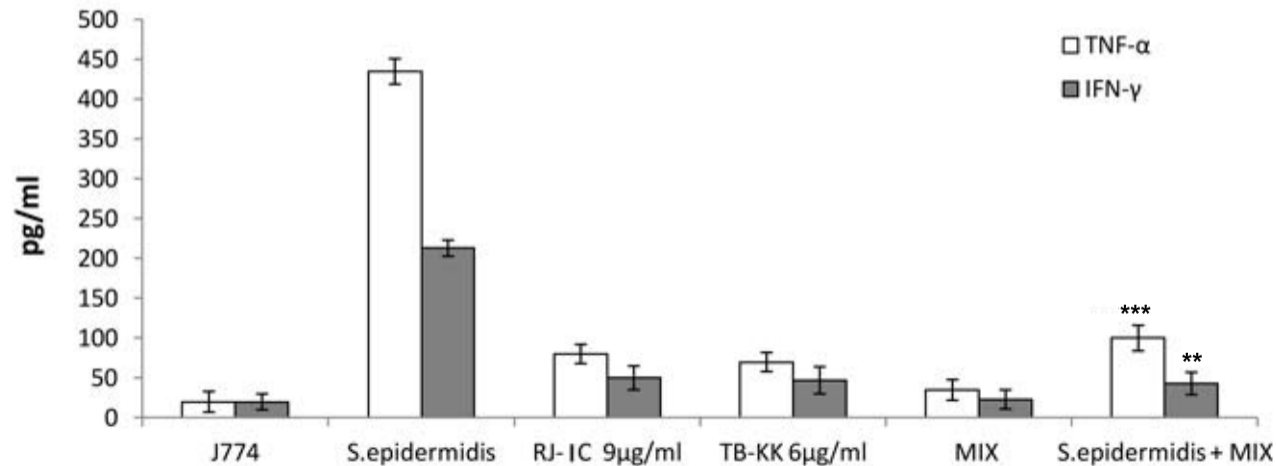

Supplement: Additional file 2 — Figure S1. Anti-inflammatory activity. The levels of IFN- γ and TNF- α were determined by a sandwich ELISA test in J774 cells untreated; J774 cells infected with S. epidermidis for 1 h; J774 cells stimulated with RJI-C (9 μg/ml) for1 h; J774 cells stimulated with TB-KK. (6 μg/ml) for1 h; J774 cells stimulated with MIX (RJI-C 9 μg/ml + TB-KK 6 μg/ml) for1 h; J774 cells infected with S. epidermidis for 1 h and stimulated with MIX for 1 h. Results from two representative experiments are presented as mean value ± S.D. *P <0.05, **p < 0.01; ***p < 0.001, Student’s t test S. epidermidis vs S. epidermidis + MIX. [file 1471-2172-13-61-S2.pdf]

Figure S2

A

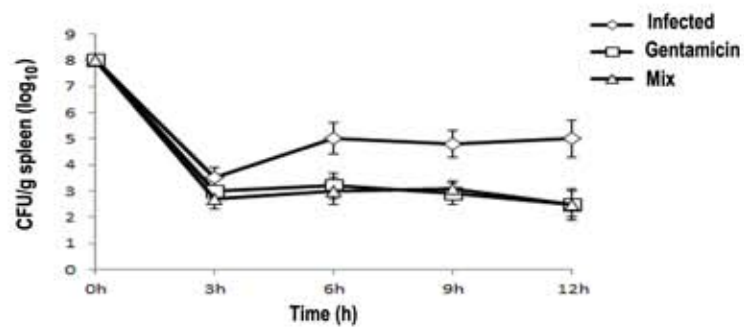

B

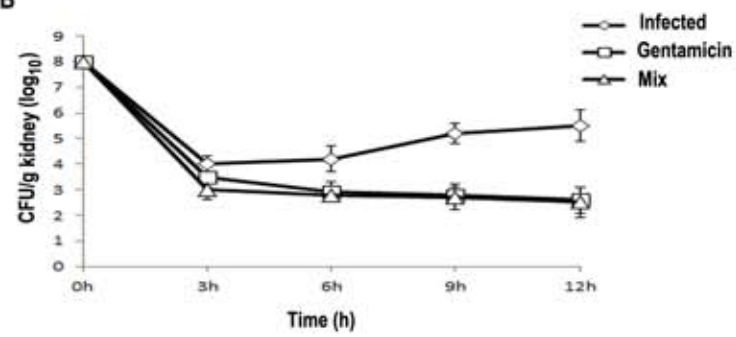

Supplement: Additional file 3 — Figure S2 (A-B). Bacterial load in spleen and kidneys of S.epidermidis infected mice (rumble line) and subsequently treated with the MIX (square line) or gentamicin (triangle line). Data are representative of 15 animals/group. Student’s t test gentamicin vs MIX not significant. [file 1471-2172-13-61-S3.pdf]

**Figure S3**

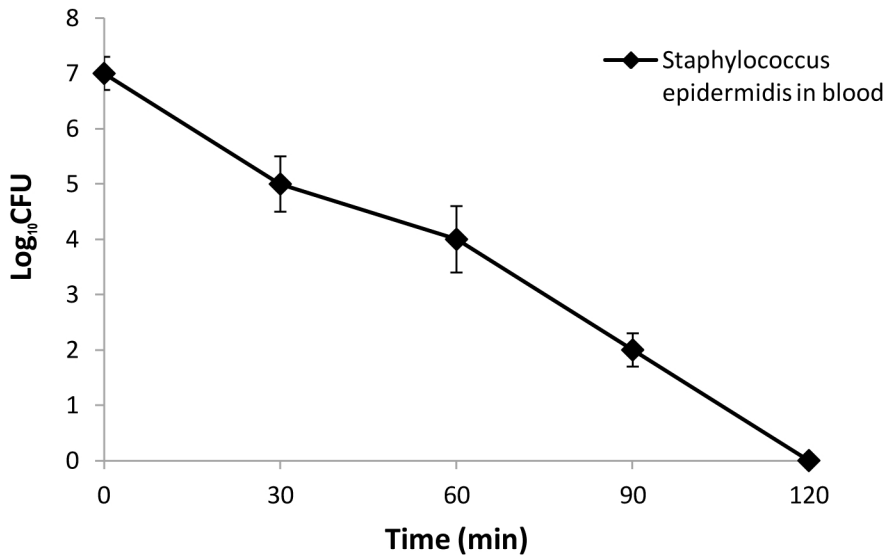

Supplement: Additional file 5 — Figure S3. Time course (30, 60, 90 and 120 minutes) of bacterial load in blood of S. epidermidis infected mice. [file 1471-2172-13-61-S5.pdf]
