# Supplementary material for: New perspectives for natural antimicrobial peptides: application as antinflammatory drugs in a murine model
Source: BMC Immunol. 2012 Nov 17;13:61. doi: 10.1186/1471-2172-13-61 (PMC3526545; doi:10.1186/1471-2172-13-61)
Supplement: Additional file 4 — Table S2. Acute phase proteins. Acute phase proteins from blood samples of mice infected with Staphylococcus epidermidis and treated with MIX or with Gentamicin. [file 1471-2172-13-61-S4.doc]

**Table S2: Acute phase proteins.** Acute phase proteins from blood samples of mice infected with *Staphylococcus epidermidis* and treated with MIX or with Gentamicin.

|  | **Naive** | **Control**  **3h** | **MIX**  **3h** | **Gentamicin**  **3h** | **Control**  **6h** | **MIX**  **6h** | **Gentamicin**  **6h** | **Control**  **9h** | **MIX**  **9h** | **Gentamicin**  **9h** |
| --- | --- | --- | --- | --- | --- | --- | --- | --- | --- | --- |
| **SAA mg/dL** | 6±0.8 | 17±0.6 | 18±1.2 | 20±0.8 | 19.1±0.5 | 16.2±0.9 | 18.4±1.2 | 21.7±0.9 | 15±0.6 | 16±0.7 |
| **Haptoglobin mg/dL** | 120±37 | 292±22.3 | 273±36.4 | 278±42.9 | 329±55.2 | 250±27.9 | 264±58.3 | 353±69.2 | 234±37.9 | 244±42.5 |
| **Fibrinogen mg/dL** | 290±44 | 407±54.2 | 370±62.3 | 385±45.2 | 422±64.3 | 345±15.9 | 364±12.6 | 457±25.8 | 311±65.5 | 340±41.0 |
