# Supplementary material for: New perspectives for natural antimicrobial peptides: application as antinflammatory drugs in a murine model
Source: BMC Immunol. 2012 Nov 17;13:61. doi: 10.1186/1471-2172-13-61 (PMC3526545; doi:10.1186/1471-2172-13-61)
Supplement: Additional file 6 — Table S3: Origin of S. epidermidis strains. [file 1471-2172-13-61-S6.doc]

**Table S3: Origin of S. epidermidis strains.**

| **Strains** | **Hospital Division** | **Specimens** |
| --- | --- | --- |
| SE | Dermatology | Skin swab |
| 3/28 | Local Health Authority | Vaginal exudate |
| 2/2 | Local Health Authority | Vaginal exudate |
| 5/6 | Nephrology | Hemoculture |
| 5/8 | Intensive care | Urineculture |
| 12/14 | Pediatrics | Hemoculture |
| 9/1 | Blood transfusion center | Urineculture |
| 10/28 | Pediatrics | Hemoculture |
| 12/26 | Emergency Medicine | Hemoculture |
| 5/25 | Medicine | Expectorate |
