# Supplementary material for: New perspectives for natural antimicrobial peptides: application as antinflammatory drugs in a murine model
Source: BMC Immunol. 2012 Nov 17;13:61. doi: 10.1186/1471-2172-13-61 (PMC3526545; doi:10.1186/1471-2172-13-61)
Supplement: Additional file 7 — Table S4: Sequences of the primers. [file 1471-2172-13-61-S7.doc]

| **Primers** | **Sequence** |
| --- | --- |
| TNF−α forward | 5’TCTCAGCCTCTTCTCATTCCT3’ |
| TNF-α reverse | 5’GTCTGGGCCATAGAACTGATG3’ |
| IFN-γ forward | 5’AGCGGCTGACTGAACTCAGATTGTAG3’ |
| IFN-γ reverse | 5’GTCACAGTTTTCAGCTGTATAGGG3’ |
| IL-10 forward | 5’ATGCTGCCTGCTCTTACTGAC3’ |
| IL-10 reverse | 5’CCCAAGTAACCCTTAAAGTCC3’ |
| COX2 forward | 5’TGTTTGCATTCTTTGCCCAG3’ |
| COX2 reverse | 5’TGAACCCAGGTCCTCGCTTAT3’ |
| Gapdh forward | 5’TTCACCACCATGGAGAAGGC3’ |
| Gapdh reverse | 5’GGCATGGACTGTGGTCATGA3’ |

**Table S4: Sequences of the primers**
